# “We give them threatening advice…”: expectations of adherence to antiretroviral therapy and their consequences among adolescents living with HIV in rural Malawi

**DOI:** 10.1002/jia2.25459

**Published:** 2020-03-02

**Authors:** Rose Burns, Denview Magalasi, Philippe Blasco, Elisabeth Szumilin, Estelle Pasquier, Birgit Schramm, Alison Wringe

**Affiliations:** ^1^ Epicentre Paris France; ^2^ Médecins Sans Frontières Lilongwe Malawi; ^3^ Médecins Sans Frontières Paris France; ^4^ London School of Hygiene and Tropical Medicine London United Kingdom

**Keywords:** adherence, adolescents living with HIV, antiretroviral therapy, Malawi, qualitative

## Abstract

**Introduction:**

Many adolescents living with HIV in sub‐Saharan Africa struggle to achieve optimal adherence to antiretroviral therapy (ART), but few studies have investigated how their treatment‐taking decisions are influenced by their social interactions with providers, caregivers and community leaders. This study aims to explore the narratives that define expectations of adherence to ART among adolescents living with HIV in a rural Malawian setting.

**Methods:**

Overall, 45 in‐depth interviews were conducted in 2016 with adolescents living with HIV, caregivers, health workers and community leaders, and four group sessions using participatory tools were undertaken with adolescents. Interviews and group sessions were audio‐recorded, transcribed and translated into English. Data were coded inductively and analysed thematically.

**Results:**

Adolescents were given strict behavioural codes around optimal treatment adherence, which were often enforced through encouragement, persuasian and threats. In HIV clinics, some staff supported adolescents with broader concerns relating to living with HIV, but other measures to address sub‐optimal adherence in HIV clinics were perceived by patients as punitive, including pill‐counts and increased frequency of clinic visits. Community leaders felt responsible for young peoples' health, sometimes attempting to influence their treatment‐taking by threatening to withdraw services, or to publically “out” those deemed to be non‐adherent. At home, discussions with adolescents about HIV were often limited to dose reminders, and some caretakers resorted to physical punishment to ensure adherence. While some adolescents complied with strictly‐enforced adherence rules, others demonstrated resistance by hiding missed doses, secretly throwing away drugs, or openly refusing to take them.

**Conclusions:**

The potential of young people to adhere to their ART may be undermined by restrictive messages and punitive approaches to enforce and control their engagement with treatment at home, in the clinic and in the wider community. Interventions should focus on creating safe spaces for adolescents to speak frankly about the adherence challenges that they face and support for caregivers including home‐based interventions.

## 1 Introduction

Adolescents are the face of a burgeoning HIV epidemic in sub‐Saharan Africa, with an estimated 1.6 million 10‐ to 19‐year olds living with HIV by 2018 1. Adolescents and young people are less likely to undergo HIV testing than adults 2, and are more likely to die, or drop out of HIV care programmes 3, 4. A systematic review of antiretroviral therapy (ART) adherence among young people aged 12 to 24 years of age estimated that only an average of 62% achieved “optimal” adherence of 95% or more 5, while virological suppression rates at 12 months after ART initiation among adolescents living with HIV (ALHIV) aged 10 to 19 years old have been estimated to range from 27% to 89% in different settings, reflecting sub‐optimal adherence histories 6.

A growing body of evidence from qualitative studies is enhancing our understanding of the experiences of HIV care engagement from the perspectives of ALHIV and their caregivers in African settings 7. This research suggests that adolescents may miss doses of their ART drugs if taking them may result in inadvertent disclosure to peers, school friends and household members. The availability or absence of social support, including from their family, peers, teachers and health workers has also been identified as a key factor that influences adherence behaviour 8, 9, 10, 11. These findings highlight the overwhelmingly social nature of the concerns that shape adolescents' adherence, suggesting that effective interventions should explicitly account for the ways in which pill‐taking is a socially situated event within young peoples' lives.

Although treatment‐taking usually occurs in the home, or at boarding schools, most studies exploring adherence to ART among ALHIV have been undertaken in clinical settings, often focusing on the underlying influence of interactions with health workers within the clinic environment, sometimes mediated through the presence of their caregiver 10, 12, 13. Bernays et al. have examined how these social interactions can undermine the capacity of young people to speak frankly about challenges they face incorporating pill‐taking into their daily lives. Furthermore, young people may experience stigmatization within the clinic setting if they miss doses of ART, because their behaviour represents a deviation from the rules and expectations of “perfect adherence” associated with biomedical notions of drug compliance 14. ALHIV may respond by feeling anxious, guilty or fearful of a health worker's reaction if they are unable to fulfil the pill‐taking instructions impressed on them during consultations, further undermining their treatment adherence.

Although several studies in sub‐Saharan Africa have explored how adolescents' social behaviours are shaped by norms transmitted in the home by their parents and caregivers, and through their interactions with peers and influential members of the community 15, 16, 17, only some have considered how these exchanges, particularly in the home environment or community setting, shape the behaviours and decisions of those who are living with HIV 18. Some research has highlighted the moral and instructive nature of messages given to ALHIV by their parents with regards to relationship formation, HIV status disclosure and treatment adherence, with these restrictions intending to protect the young people and their families from the perceived consequences of HIV status disclosure 19. However, there has been less exploration of the consequences of these interactions on young people's ART adherence. In view of this knowledge gap, we aimed to explore how social narratives and interactions within the everyday lives of ALHIV shaped their adherence behaviours.

## 2 Methods

### 2.1 Study context

This study took place between June and August 2016 in Chiradzulu, a rural district of Malawi with an HIV prevalence of 17% and a population of approximately 288,000 19. Médecins sans Frontières supported the Ministry of Health in providing HIV care and treatment in the eleven district‐level health facilities since 2001. By 2016, approximately 33,000 patients were receiving ART in this programme, of whom 1700 were adolescents aged 10 to 19 years of age, with the majority perinatally infected. Routine programme data showed that 32% experienced virological failure (VL ≥ 1000 copies/mL) in 2016. This qualitative study took place alongside a cross‐sectional assessment measuring virological failure, drug resistance and treatment adherence among 10 to 19 year olds on first‐line ART.

Social organization in Chiradzulu is hierarchical with power concentrated in the traditional authority system and the state, the two of which are integrated in Malawi. “Village chiefs” are the official link between communities and the state, mediating interactions with various actors, overseeing community‐based government initiatives such as community AIDS committees, and controlling allocation of land and distribution of food aid 20.

### 2.2 Data generation

Repeat in‐depth interviews (IDI) were held with sixteen ALHIV, and four group activities incorporating participatory learning and action (PLA) tools were undertaken with ALHIV. IDI were also held with sixteen caregivers of ALHIV (including caretakers of participating adolescents), six health workers, and seven community members seen as influential to young peoples' behaviour (Table 1).

**Table 1:** Study participants

Characteristics	Tool and category	Sex		Totals
	IDIs	M	F	
**1. ALHIV (repeat interviews)**				
Age (years)	10‐13	4	3	7
	14‐16	3	1	4
	17‐19	1	4	5
Viral load	≥ 1000 copies/ml	7	3	10
	<1000 copies/ml	1	5	6
***Total ALHIV***		***8***	***8***	***16***
**2. Caregivers of ALHIV**				
Disclosure of	Caregivers of fully disclosed ALHIV	2	6	8
ALHIV	Caregivers of partially disclosed ALHIV	2	6	8
Type of caregiver	Parent	2	7	9
	Other relative	2	4	6
	Other caregiver	0	1	1
***Total caregivers***		***4***	***12***	***16***
**3. Community persons of influence**				
Role	Traditional and religious authorities	4	0	**4**
	Youth leader/sports coach	2	0	**2**
	Teacher	1	0	**1**
***Total community persons of influence***		***7***	***0***	***7***
**4. Health workers**				
Role	Clinical staff	3	0	**3**
	Counsellors and support staff	2	1	**3**
***Total health workers***		***5***	***1***	***6***
	**Total IDI participants**	**24**	**21**	**45**
**Group activities using PLA tools**		**M**	**F**	**Total**
	Adolescents living with HIV (2 groups)	4	7	11
**Total PLA participants**		***4***	***7***	***11***
**Total participants (IDI + PLA)**		**28**	**28**	**56**

All tools were piloted and adapted accordingly. Initial interviews with ALHIV focused on developing rapport with the participant and understanding their life histories, while the second interview focussed on adolescents' lived experiences of HIV care‐seeking and treatment‐taking based broadly on a socio‐ecological framework 21 and incorporating a vignette to encourage discussion of adherence challenges 22. PLA tools included community mapping, which involved ALHIV drawing places of importance to them in their community, and body mapping where they drew themselves and health workers, highlighting different characteristics. “Problem tree” analysis were also used to elicit some of the perceived causes and consequences of adherence problems from the adolescent perspective 23. Following “ice‐breaker” games, adolescents undertook the activities in small groups, with the resulting drawings then presented to the group and used to stimulate discussion. These tools were used to elicit prevalent views among adolescents about HIV and ART in a format that engaged young people. Interviews with adults (caregivers, health workers and community members) covered their relationships with ALHIV, perspectives and experiences with HIV services, and community attitudes towards adolescents and HIV. Data were also generated through observations carried out at HIV clinics, youth clubs and at social events attended by young people. Observations were recorded into templates by fieldworkers, and included content of health promotion messages, the physical clinic environment, interactions between patients and providers, and services provided to young people. Structured observations of the interview setting (including participants' living arrangements) were also written up and discussed to support analysis of each transcript. Data were collected by two locally recruited non‐clinical fieldworkers with social science qualifications who were not previously known to participants. In order to promote rapport with the participants, these fieldworkers were young adults, matched to participants' gender. Health worker interviews were also conducted by the lead author (RB).

### 2.3 Sampling strategy

Adolescents and their caregivers were purposively sampled from participants in the cross‐sectional assessment, which included patients aged 10 to 19 years who were on first‐line ART for at least six months. Using data from the cross‐sectional assessment, we generated a sampling frame from which we excluded ALHIV who had not gone through full HIV and ART disclosure. We used an intensity sampling approach 24 to identify information rich cases, ensuring diversity in terms of virological outcomes, age, sex and type of health facility, as these characteristics may shape adherence experiences.

We continued sampling participants until we had reached saturation with regards to key themes that emerged during the analysis process. Since ALHIV with the most apparent adherence challenges and/or virological failure were often those who found it most difficult and challenging to provide longer descriptions of their circumstances, this led to a relatively high number of ALHIV with virological failure in our sample.

Caregivers from the sampling frame were selected to include a range of caregiving circumstances (parents, other caregivers) and to include (i) caregivers of adolescents who had undergone full disclosure and who were also participating in the IDI; and (ii) caregivers of adolescents who had gone through a partial disclosure process (Table 1). Recruitment for group activities took place through HIV peer support groups for ALHIV to ensure participants had disclosed their status to the group. Health workers were purposively sampled to ensure a range of cadres providing services to adolescents. Community members were selected based on PLA activities that mapped places and people of importance to young people (for example football clubs and coaches).

### 2.4 Data management and analysis

Interviews and PLA activities took place in Chichewa (or in the case of some health workers, in English), and were recorded, transcribed and translated into English. All quotations presented are from IDIs unless the notation specifies PLA. Photographs were taken of PLA group drawings. Transcripts, study diaries and notes on photographs were coded by the lead author (RB) inductively and iteratively using Nvivo11. The content of the interviews and observations made during the interview process were discussed daily between the fieldworkers and lead author (RB), and weekly between the lead author and senior author during the data collection, coding and analysis periods in order to ensure that multiple perspectives and interpretations were considered by RB who led the coding and analysis for this manuscript. The visual data were analysed collectively during meetings with the study team. Throughout the analysis process, we prioritized the meaning that participants gave to their experiences of living with HIV 25. Coding was initially descriptive, with emerging concepts identified and named, and relationships between codes regularly discussed between the researchers and fieldworkers. “Deviant” cases were sought in the data to refine our understanding of the relationships between the emergent themes and the concept of dyadic analysis was drawn upon to compare accounts in those cases of adolescent‐caretaker “pairs” recruited 26, 27. Analytical memos were used to enable reflections on the relationships between higher order concepts which were documented as they emerged.

### 2.5 Ethics approval

Ethics approval was granted by the Malawian National Health Sciences Research Committee (reference 15/7/1446) and the London School of Hygiene and Tropical Medicine (reference 10003). Written informed consent was obtained from all participants 18 years and over. For participants under 18 years of age, written informed consent was sought from the caregiver, with assent obtained from the adolescent.

## 3 RESULTS

Many ALHIV talked about the importance of taking their drugs regularly to preserve their health, and most expressed a desire and willingness to take them as prescribed. However, some young peoples' intentions to take their pills were undermined by the challenges that they faced in trying to adhere to the strict conditions that accompanied pill‐taking which were imparted in the context of their clinic visits, in their communities or at home, as described below:

### 3.1 “Health workers should also show love to us”: adherence conversations within HIV clinics

In describing their interactions with health workers at the HIV clinics, many ALHIV recounted the standardized instructions that they received with regards to taking their drugs. Most ALHIV had been given a twice‐daily prescription, with one dose taken at six o' clock in the morning, and another at six o' clock in the evening, with the importance of the precise timing of the doses strongly emphasized. Some participants appealed for tailored approaches to timing of doses to suit their personal circumstances, as well as more open and supportive dialogue with health workers around their well‐being and pill‐taking:

*Health workers should be friendly like explaining medication and what we can do in various situations…health workers should also show love to us (adolescent female living with HIV, PLA activity)*

**Figure 1:**
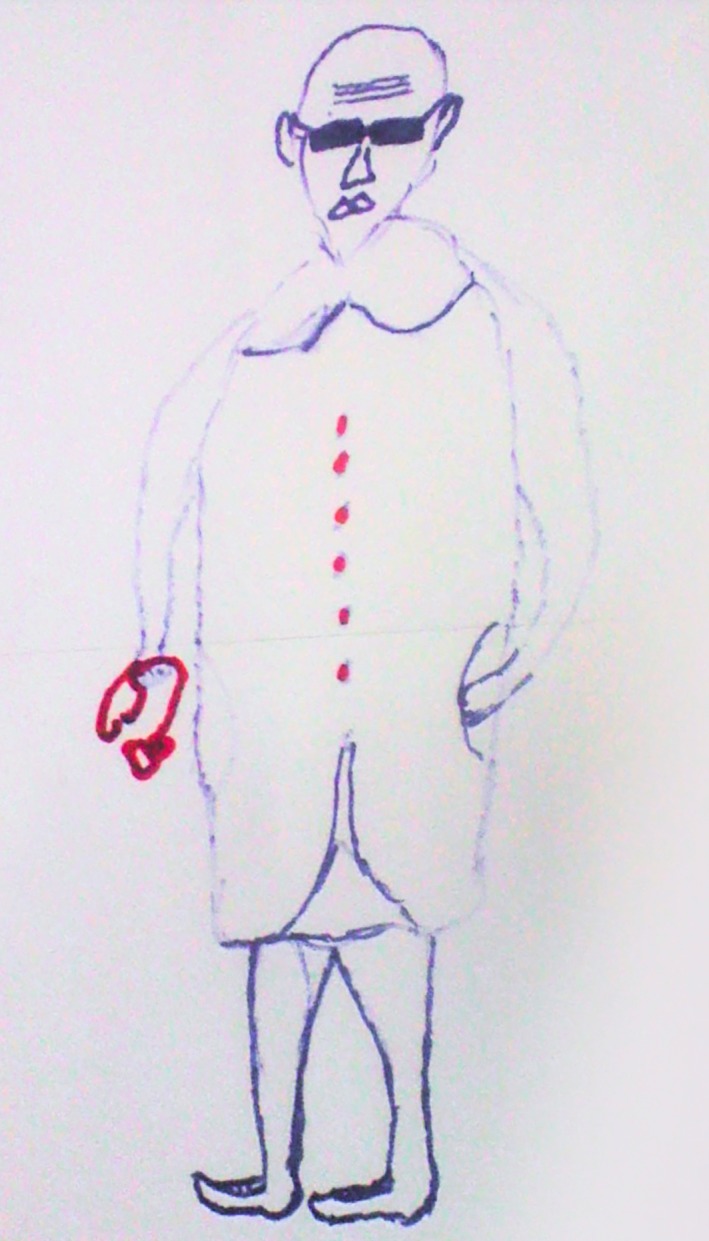
Depiction of a clinician drawn by ALHIV during PLA group activities.

Other young people described how discussions around the everyday challenges of adherence were sometimes discouraged by health workers who could adopt aggressive or angry tones during their interactions, particularly if they were busy. In one of the PLA group activities with ALHIV, the participants depicted a doctor described as fat and wearing expensive sunglasses (to reflect participants' perception of the doctor's corruption), frowning and bad‐tempered with patients (Figure 1), with one participant explaining:

*Other doctors are better off, but the kind we have drawn, are those ones that cannot manage to stay for 10 minutes while explaining to them about your problems. They would even shout at you for delaying them…(adolescent male living with HIV, PLA activity)*

There were also multiple examples of young people, or their caregivers, being scolded when they did admit to missing doses of their medication:

*Sometimes health workers say that rudely… like I have been observing for some time, especially to people who miss taking the medicine, and those who miss the appointments. So they get shouted at, like: ‘What were you doing?! Do you think you are normal and not sick?!' (Caregiver)*

Some measures that were adopted by health workers to promote adherence could be perceived by young people as punitive, including the use of pill‐counts to check adherence. Extra appointments, or longer waits to be seen at the clinic were also sometimes handed out to those whose adherence behaviours did not align with the health workers' expectations as one participant explained:

*If you come and get medication for two months in advance, they would deliberately give you medication worth only a month so as to punish you …so we end up wasting transport money and other costs … (adolescent male living with HIV)*

“Good” behaviour among young people was often rewarded with some health workers using incentives, persuasion and encouragement to promote adherence. Many patients reported this as supportive. For example, counsellors often tried to instil into young people the desire for a healthy body as a way to motivate them to keep taking their drugs. Some health workers even went beyond their clinic roles to try and find ways to provide moral or practical support to help their patients to adhere to treatment, such as providing snacks in the clinic queues or school books for their education. Some participants responded by trying to improve their adherence in acknowledgement of the respect that they felt had been accorded, and through a desire not to disappoint the health workers with whom they had developed a positive rapport:

*We had to miss an appointment to refill the medicine with the girl and we told the doctor who was not mad but rather said in a lovely way that he was not really happy with our act. So since then we started thinking that… we should pay him back by following all the procedures. The doctor just wanted what's good for our child…(Caretaker)*

### 3.2 “We prohibited those activities”: community leaders as moral guardians of young people

Community leaders often saw themselves as playing an important role in terms of providing moral guidance to young people, including with regards to adherence to ART. Various youth activities were monitored by community leaders, and some of the techniques that were adopted to ensure adherence to ART, including encouragement, persuasion and threats, mirrored those used by health workers. In particular, some community leaders were concerned about perceived temptations for young people to drink or take drugs which they felt lead to young people becoming non‐adherent, or sexually active and HIV‐infected:

*As for alcohol…..we gave them rules that the sellers should sell, and people should drink until 6 o'clock. If 6 o'clock passes…they should buy and take to their homes. We prohibited those activities whether initiation ceremonies and others, we started to outlaw them that no, these things are damaging our culture in accordance with this disease, that's where harmful advice is given and people pay fines of goats or chickens. (Community leader)*

Other community leaders established forums or activities to keep young people occupied with more socially acceptable activities such as sport or choir singing. In one instance, after‐school football matches that allowed boys and girls to meet were stopped in a bid to reduce opportunities for sexual encounters between young people. Breaking community rules that were intended to control HIV was reported to incur fines of livestock or chickens that were imposed by village leaders. In another case, a community leader suggested that health centres provide lists of non‐adherent patients to traditional authorities so that they could be publicly stigmatized regarding their HIV status at community meetings as a punishment and deterrent to other young people. Others suggested that non‐adherent patients could be threatened with a ban from attending further appointments at the clinic and would not be aided with support letters for referral to health and other services:

*There are others who when they take the medicine and see that they are good, they stay without taking medication, they just stop. So when we find such things we give them a threatening advice that if they do that, they won't be received at the hospital and also we won't write those letters…(Community leader)*

### 3.3 “Granny could chase and whip me”: Enforcing adherence rules at home

The home emerged as a key place where adherence behaviours were enacted as drugs were almost always taken at home in the mornings and evenings. While many caregivers reported supportive relationships with their children, their encouragement to adhere was often restricted to reminders to take doses at the required times. Caregivers rarely engaged in conversations about how transmission had occurred, or about living with HIV more broadly, reinforcing it as shameful, stigmatizing and something best kept secret. When discussions around HIV and ART did occur, they were often directive and without explanations, as one community leader indicated:

*On the side of the parents, they do not pay attention to what the child says, and there is no dialogue between them, as the parents only issue commands to the children (Community leader)*

In some cases, incitements to adhere to ART by caretakers were underpinned by threats of the dire consequences of failing to take doses, including treatment failure or death, which were then also reflected in the adolescents' own accounts of these risks:

*I just told him that the medicine is supposed to be taken everyday otherwise one dies…so I think that in a way scared him which is why he has never forgotten, he [even] sometimes reminds me to take mine when I forget to (Caregiver).*

*If you don't adhere, you help the virus to get stronger. So, in her case she conforms to bad behaviour and doesn't take her pills while destroying her life. She is ruining her future (adolescent girl living with HIV, PLA activity)*

Several young people reported being harshly disciplined or being physically punished if they were perceived to have contradicted their caregivers' instructions relating to taking their HIV drugs:

*…At Granny's she could see what was happening, when I threw the medicine away Granny could chase and whip me (adolescent male living with HIV)*

*When I am wrong, then I am beaten up.*

* Who beats you? *

*My sister (adolescent female living with HIV)*

Unsurprisingly, some adolescents gave contradictory descriptions of their home life than those provided by their caretakers who may not directly discuss physical punishment. For many caretakers, discipline took place in the context of overwhelming challenges such as economic hardship, caretakers were stressed and faced competing priorities attempting to support an adolescents' HIV care:

*Sometimes [name of adolescent female] even misses her dose because of a lack of food. She also complains of hunger due to taking the drugs, so if we have no food she cannot take medication. But we try to force her to do so, and she thinks we are mean (caregiver)*

Caretakers' also used encouragement and rewards to ensure that ALHIV engaged with treatment expectations:

*Sometimes they would lie to me that they will buy a bicycle…I continued taking the medicine and when I grew up asking them about the bicycle they tell me that it was just a way of encouraging me to take my medicine (adolescent male living with HIV )*

### 3.4 Adolescents' responses: “I would just shake the bag….”

Faced with rigidly enforced social expectations in their home lives and interactions in the clinic and community, adolescents responded in a variety of ways. Some young people conformed or felt motivated by the guidance that they received from the adults in their lives, often linking these interventions with their determination to take their pills as prescribed, or to face stigmatizing experiences or address relationship concerns:

*I would like to find a man, but he has told me that we should go and get tested: so I ask her [the health worker] ‘what should I do'? She advised me that I should get help from a counsellor. And if there's problems with people laughing at me, taunting me, I come to tell her and she says I shouldn't be worrying (adolescent female living with HIV)*

Adherence was sometimes encouraged through open and frank conversations around disclosure or reasons for adhering to treatment, which could prompt more regular pill‐taking:

*…there was a concern that my performance in football was getting low. So when I understood and they disclosed [my HIV status] to me then my performance started increasing…it [my performance] was down because at that time I was thinking much about it [HIV]. So when my parents talked to me to take it as a normal thing I released my strength (adolescent male living with HIV)*

However, disciplinary and punitive actions were often counterproductive, with some young people resisting taking their pills, and then hiding the fact to avoid being reprimanded. For example, some adolescents described subverting adherence expectations by inconspicuously throwing pills away in the garden, or pretending to take their doses:

*Like when I come late I would find everyone asleep …so sometimes I would take and sometimes I would not take [ART], sometimes I would just shake the bag so that they would think that I have taken..when I had not taken anything. (adolescent male living with HIV)*

For some young people, the choice to swallow their pills or not also represented an expression of agency to make a decision, take control of their life or express their discontentment with a social situation:

*when she is angry, she was not taking the drugs. And when her relatives are forcing her to get married, she was not taking the drugs.. And [now] she is on second line ART…(health worker)*

## 4 Discussion

This study explored the social interactions that shape the pill‐taking behaviours of young people living with HIV in a rural Malawian setting. We found that adolescents demonstrated good understanding of the importance of regularly taking their drugs, and usually wanted to adhere to their treatment. However, these intentions were often undermined by their inability to conform to strict treatment rules and requirements that were enforced by health workers, family members and community leaders, sometimes using punitive measures.

Our findings align with those of other studies that suggest that the choices that adolescents make in relation to health‐related behaviours, including pill‐taking, are frequently restricted by the “moral, social and cultural consent” of the adults in their lives 14, and the messages that they impart 28. As such, their agency to enact health‐promoting behaviours is “bounded” by their placement within the lower echelons of the hierarchies that exist within their families, in HIV clinics and within the communities in which they live 29. Our findings suggest that while some young people respond to strict instructive messages by adhering to their medication, others demonstrate resistance through acts of defiance such as refusing to take their medication or employing various ruses to give the impression of having done so.

In our study, ALHIV chose to hide missed doses for a host of reasons including as a means of self‐protection from disciplinary or punitive measures. Our findings accord with other studies that suggest that ALHIV may under‐report non‐adherence so as not to disappoint clinical staff and counsellors with whom they may have formed close or dependant relationships 30 and as a strategy to avoid the stigmatization associated with less than perfect adherence 10, 14. Clinical protocols and counselling messages need to convey the importance of optimal adherence while acknowledging that “perfect” adherence may not be needed for viral suppression 31 even if the nuances of this are challenging to communicate to patients, and that virological failure due to drug resistance cannot be resolved through improved adherence to the same regimen 32. A reasonable margin of error in the timing of doses may reduce adolescents' secrecy and ultimately improve their treatment adherence 14, 28.

Our findings accord with those from studies with ALHIV in Zimbabwe and Uganda which highlighted the limitations of treatment literacy approaches which focused on imparting medical knowledge regarding the relationship between HIV, ART and physical health, despite their ongoing prominence in HIV care guidelines for ALHIV 12. Counselling sessions should be seen as an opportunity to address the array of concerns facing ALHIV which may impinge on their adherence, in a non‐judgemental manner, as they navigate the transition towards adulthood. These sessions should allow ALHIV to take ownership of medical information through meaningful dialogue, make sense of it within their worldviews and develop problem‐solving strategies for addressing life challenges as well as adherence in day‐to‐day situations 33, 34.

Most studies exploring adherence among adolescents focus on the clinic setting (e.g. 35) with resulting recommendations typically focused on clinic‐based activities that target young people through peer‐led or health worker‐led interventions 36. While these are likely to benefit ALHIV, our findings suggest that additional approaches that address the underlying social, economic and structural determinants of behaviour among ALHIV as well as the wider community, and which have been used to effectively inform community mobilization efforts around HIV, should be explored 33. Most community leaders and health worker participants were male, reflecting their gender distribution in this setting. This gender disparity indicates that men may be dominant in terms of establishing norms around adherence that are communicated in community and health facility contexts in this setting. We found that the adolescent males in our study tended to give more detailed, longer answers during the interviews, resulting in richer data for use in illustrative quotations. “Silences” when recounting HIV and ART experiences may reflect social norms which disadvantage self‐expression among adolescents, and particularly among females, as well as instructions from caregivers to remain silent about their condition 30, 37. These silences highlight the relative disempowerment and limited agency of adolescents, especially among females, in discussing this aspect of their lives 14. In addition, our findings also align with a growing understanding that some caregivers need additional support to care for ALHIV 38, 39, and that such interventions may be best delivered outside of the clinic, through home visits 40, 41, 42, 43, 44. Such initiatives need to acknowledge the inter‐generational conflicts that are often commonplace within homes, and that parenting interventions beyond those focusing on adherence may be needed 45. Based on these findings, several interventions have since been implemented in Chiradzulu, including home visits from counsellors to support caretakers and patients with adherence challenges, and clinic based interventions such as health worker training and “teen clubs” emphasizing constructive dialogue between adolescents, caretakers and health workers about their treatment. Evaluation of the impact on various outcomes is ongoing.

Our findings need to be considered in relation to the inherent challenges of adults interviewing ALHIV about their treatment‐taking behaviours. We used repeated IDIs to build up rapport with our adolescent participants, ensured that fieldworkers were young adults to minimize the generation gap, and carried out extensive fieldworker training. We additionally incorporated the use of vignettes which can be valuable for eliciting participants' perspectives on sensitive topics 22. An important strength of our study was our ability to draw on multiple participant groups' perspectives on issues underlying young people's ART adherence, and photographs and documents which did not rely on the spoken word, and which provided nuanced descriptions of the tensions that may underlie their missed doses. Our findings concern ALHIV who were perinatally‐infected and receiving first line ART, and may differ from those infected through sexual transmission or ALHIV taking other regimens. Further research should be conducted to understand treatment‐taking experiences among ALHIV with other defining characteristics.

## 5 Conclusions

In conclusion, we found that some ALHIV in this setting had the knowledge, but lacked the agency, to adhere to their ART drugs in the face of strictly enforced timings and prescribed pill‐taking conditions. Punitive and controlling techniques that were sometimes used by health workers, community leaders and family members to enforce adherence may undermine treatment engagement for many young people, and should be replaced by interventions that enable ALHIV to participate in open, meaningful and supportive dialogue that helps them to incorporate regular pill‐taking into their daily lives.

## Competing interest

The authors declare that they have no conflicts of interest.

## Authors' contributions

AW, BS and ES conceived the study and contributed to its design. DM and RB undertook data collection and fieldwork. AW, RB and DM, undertook the data analysis, and AW, RB, DM, PB and EP interpreted the data. AW and RB co‐wrote the first draft of the manuscript. All authors reviewed, edited and approved the final manuscript.
